# The UA Doppler Index, Plasma HCY, and Cys C in Pregnancies Complicated by Congenital Heart Disease of the Fetus

**DOI:** 10.3390/jcm11195962

**Published:** 2022-10-10

**Authors:** Xiaona Xu, Baoying Ye, Min Li, Yuanqing Xia, Yi Wu, Weiwei Cheng

**Affiliations:** 1 Prenatal Diagnosis Center, The International Peace Maternity and Child Health Hospital, School of Medicine, Shanghai Jiao Tong University, Shanghai 200030, China; 2 Shanghai Key Laboratory of Embryo Original Diseases, Shanghai 200030, China; 3 Department of Ultrasonography, The International Peace Maternity and Child Health Hospital, School of Medicine, Shanghai Jiao Tong University, Shanghai 200030, China; 4 Shanghai Municipal Key Clinical Specialty, Shanghai 200030, China

**Keywords:** congenital heart disease, homocysteine, cystatin C, the UA Doppler index

## Abstract

Background: Congenital heart disease/defect (CHD) is one of the most common congenital disabilities. Early diagnosis of CHD can improve the prognosis of newborns with CHD. The aim of this study was to evaluate the relationship between the factors and the onset of fetal congenital heart disease by measuring fetal umbilical artery (UA) Doppler index, maternal HCY, and Cys C levels during pregnancy. Methods: This retrospective study analyzed 202 fetuses with CHD, including 77 cases (39.1%) of simple CHD and 120 cases (60.9%) of complex CHD. Singleton pregnant women who were examined at the same time and whose malformation screening did not suggest any structural abnormalities in the fetus were assigned to the control group (*n* = 400). The UA Doppler index, plasma HCY, and Cys C levels were compared among the pregnant women across the three groups, and logistic regression analysis was performed on statistically significant markers. The ROC of UA S/D, PI, RI, HCY, and Cys C were plotted, and the area under the ROC (AUC) was calculated. Results: The UA S/D, PI, and RI in the complex CHD group were significantly higher than those in the control group (*p* < 0.05). The levels of HCY and Cys C in the CHD group were significantly higher than those in the control group (*p* < 0.05). HCY and S/D revealed a positive correlation (r = 0.157), and the difference was statistically significant (*p* < 0.001). Cys C and S/D were positively correlated (r = 0.131), and the difference was statistically significant (*p* < 0.05). The levels of UA Doppler indices, maternal plasma HCY, and Cys C were elevated in fetuses with CHD. The AUC of the combined test of the UA index, HCY, and Cys C was higher than that of each individual test. Conclusions: Elevated levels of the UA doppler indices, HCY, and Cys C during pregnancy are positively associated with the development of congenital heart disease in offspring. The combination of HCY and Cys C was the most efficient test for the diagnosis of CHD. We are the first to report that plasma Cys C levels of women pregnant with fetuses with CHD were higher than those of women pregnant with normal fetuses.

## 1. Introduction

Congenital heart disease/defect (CHD) is one of the most common congenital disabilities, with a prevalence rate of 1 in 100 live births. It is one of the leading causes of perinatal mortality [1]. With the rapid development of prenatal imaging technologies, the application of fetal echocardiogram to detect CHD has advanced, and the use of color Doppler noninvasive detection of fetal hemodynamic changes is also becoming gradually common. Several studies have reported [2,3,4] that the increase in maternal plasma homocysteine (HCY) level is closely associated with cardiac malformations in their offspring. The maternal plasma cystatin C (Cys C) is a biomarker of early kidney injury [5] and also a novel cardiac biomarker [6] independently associated with the risk of cardiovascular anomalies and mortality. The relationship between Cys C level during pregnancy and the occurrence of fetal CHD has not been reported yet. In this study, we aimed to determine the relationship between the factors and the onset of fetal congenital heart disease by measuring fetal umbilical artery (UA) Doppler index, maternal HCY, and Cys C levels during pregnancy.

## 2. Materials and Methods

### 2.1. Patient Cohort

The present study is a retrospective study. Fetal cases diagnosed as CHD by fetal cardiography in International Peace Maternity and Child Health Hospital from January 2015 to December 2021 were enrolled in the CHD group. Fetal cases with normal anomaly scans were enrolled in the control group. Electric clinical medical charts were reviewed to obtain demographic information on maternal age and pregestational body mass index (BMI). Serological screening results of maternal plasma HCY and Cys C level at 12–14 weeks of gestation were also collected from the medical charts.

The inclusion criteria were as follows: fetuses with CHD were included in the CHD group. The control group was normal fetuses without structural malformation matched with maternal age and gestational age, and a 1:2 ratio was used. The exclusion criteria were as follows: fetuses with CHD combined with extracardiac anomalies, multiple pregnancies, and pregnancies with maternal obstetric complications.

This study was approved by the Medical Ethics Committee of the International Peace Maternity and Child Health Hospital (GKLW 2019–24). Written informed consent was obtained from the pregnant women involved in our study.

CHD was diagnosed by ultrasound and clinical diagnosis. Based on the codes of the International Classification of Diseases, Ninth Revision, Clinical Modification, complex CHD (CCHD) refers to all types of CHD except for simple CHD (SCHD). The main types of SCHD included ventricular septal defect (VSD), aortic valve stenosis, pulmonary stenosis, patent ductus arteriosus, and secundum atrial septal defects The fetal heart defects that were not included in the study were persistent left superior vena cava, simple right aortic arch, aberrant right subclavian artery, and heart tumors. The main types of CCHD include tetralogy of Fallot (TOF), tricuspid atresia and stenosis, coarctation of the aorta, hypoplastic right heart syndrome, tricuspid valve dysplasia, atrioventricular septal defect, single ventricle, interruption of aortic arch, pulmonary atresia, persistent truncus arteriosus, hypertrophic cardiomyopathy, vascular ring, hypoplastic left heart syndrome, transposition of great arteries, and double outlet right ventricle [7,8].

### 2.2. Fetal Echocardiogram

The gestational age range for echocardiography was 18–28 weeks. Detection of fetal echocardiography was performed based on the Guidelines of the International Society of Ultrasound in Obstetrics and Gynecology (ISUOG) [9]. Ultrasound examination was performed by radiologists with prenatal ultrasound diagnostic qualifications using GE Voluson E10 (GE Healthcare, Zipf, Austria), Philips iU Elite (Philips, Copenhagen, Denmark), or Philips iE33 (Philips, Copenhagen, Denmark). Subjects were kept a flat, lying position and exposed their lower abdomen. The number of fetuses was routinely checked, multiple pregnancies were excluded, and the fetal orientation was determined. The transverse and four-chamber heart section of the fetal abdomen was determined along with the position of the internal organs, the heart, and the cardiac axis. Based on this, the left and right ventricular outflow tract, long axis and short axis section, three-vessel section or three-vessel tracheal section, aortic arch section, ductus arteriosus arch section, and the vein-atrial connection section determined the fetal atrioventricular connection relationship. The left and right atrioventricular valves determined the fetal ventricular–artery connection relationship. The interrelationship between the great arteries, the ratio of the inner diameter of the aortic arch and the ductus arteriosus, and finally the measurement results were collected and stored.

All fetuses with CHD were diagnosed by two experienced fetal echocardiographists. Postnatal results were obtained from the newborn’s examination records at the hospital or directly from the parents. For CHD cases, neonatal echocardiograpy was performed by another examiner (approximately 2–4 days after birth) prior to discharge. A clinical examination by an experienced pediatrician included auscultation of the heart murmur and oxygen saturation of the upper and lower extremities. The newborn/fetus was considered normal if no abnormalities were suspected or found.

### 2.3. The UA Doppler Index at 22–24 Weeks of Pregnancy

When ultrasound fetal malformation screening was performed at 22 to 24 weeks of pregnancy, after detecting the free segment of the umbilical cord, the umbilical artery blood flow spectrum was obtained. When measuring the umbilical artery spectrum, pregnant women had to hold their breath for 3–5 s to facilitate the acquisition of a stable umbilical artery spectrum. The indices of systolic/diastolic ratio (S/D), resistance index (RI), and pulsatility index (PI) were measured and recorded.

### 2.4. Blood Sampling and Biochemical Characteristics at 12–14 Weeks of Pregnancy

Pregnant women underwent fasting after 22:00 on the day before the first antenatal examination in the 12th to 14th weeks of pregnancy. A total of 3 mL of venous blood was drawn on an empty stomach at 08:00–10:00 on the day of examination. The serum was routinely separated, and the levels of Hcy and Cys C were measured. The automatic biochemical analyzer was used for detection, and the detection of various indicators was performed according to the instructions given in the kit.

### 2.5. Statistical Analysis

SPSS 25.0 statistical software was used for analysis. The measurement data following the normal distribution were expressed as a one-way analysis of variance. The data are expressed as mean ± standard deviation. The comparison of the counting data is expressed by the Wilcoxon rank sum test, and the data are expressed as frequencies. Pearson’s (r) correlation was performed to analyze the correlation between maternal plasma HCY, Cys C level, and the UA doppler indices, and the logistic regression analysis of the various influencing factors of fetal congenital heart disease was performed. A *p*-value < 0.05 indicated that the difference was statistically significant.

## 3. Results

A total of 197 fetuses with CHD were assessed, which included 77 cases (39.1%) of simple CHD and 120 cases (60.9%) of complex CHD. The type of CHD is depicted in Table 1. The demographic characteristics of the patients in the CHD groups and the healthy control groups are depicted in Table 2. The mean maternal age and pregestational BMI were not significantly different between the CHD and control groups (*p* > 0.05; Table 2).

### 3.1. The Comparison of the UA Doppler Indices at 22–24 Weeks of Pregnancy between the CHD and Control Groups

The UA S/D, PI, and RI in the complex CHD group were significantly higher than those in the control group (3.43 ± 0.84, 1.18 ± 0.19, 0.70 ± 0.06 vs. 3.21 ± 0.57, 1.12 ± 0.16, 0.68 ± 0.05; *p* < 0.05, respectively). No statistically significant difference was noted in the UA Doppler indices between the simple CHD group and the complex CHD group. In addition, no statistically significant difference was noted in the UA Doppler indices between the simple CHD group and the control group (Table 3).

### 3.2. The Comparison of Maternal Plasma HCY and Cys C Levels at 12–14 Weeks of Pregnancy

The levels of HCY and Cys C in the simple CHD group were significantly higher than those in the control group (*p* < 0.05). The HCY and Cys C levels in the complex CHD group were significantly higher than those in the control group, and the difference was statistically significant (*p* < 0.05). However, the difference in the HCY and Cys C levels between the simple and complex CHD groups was not statistically significant (Table 4).

### 3.3. Correlation between the Plasma Biochemical Indicators and the UA Doppler Indices

Pearson’s correlation was applied to analyze the maternal plasma HCY and Cys C levels and the UA Doppler indices. The results revealed that HCY and S/D had a positive correlation with PI (r = 0.157, 0.088; *p* < 0.05, respectively). Cys C and S/D, PI, and RI were positively correlated (r = 0.131, 0.118, 0.118; *p* < 0.05, respectively) (Table 5, Table 6 and Table 7).

### 3.4. Analysis of the Influencing Factors of Fetal Congenital Heart Disease

Multifactorial logistic regression equations were constructed by including age, pregestational BMI, UA S/D, UA PI, UA RI, and maternal plasma HCY and Cys C levels with the presence or absence of CHD as the dependent variable. The results showed that the effect of UA S/D, PI, RI, and the level of maternal plasma HCY and Cys C on fetal CHD were statistically significant (*p* < 0.05). The ORs for UA RI and Cys C were especially high (69.55 and 24.75) (Table 8).

**Figure 1:**
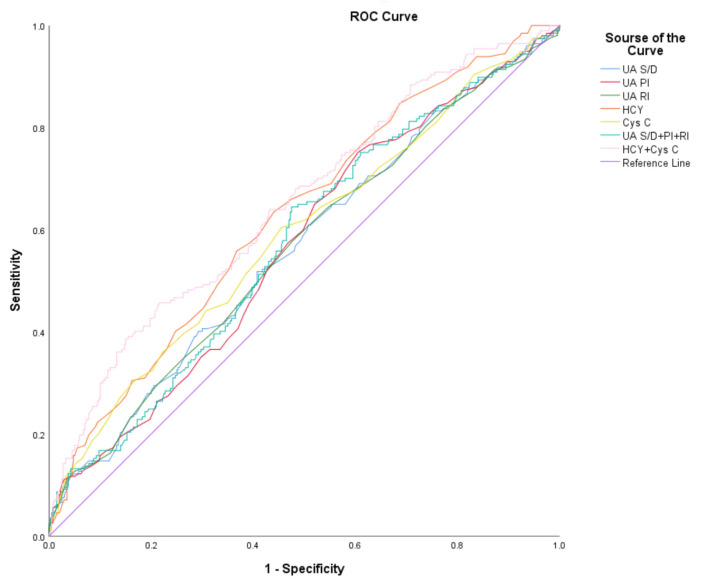
ROC Curve.

The ROC of UA S/D, PI, RI, HCY, and Cys C were plotted, and the area under the ROC (AUC) was calculated. The binary logistic regression analysis was used to obtain the cut-off value of the combined prediction probability of each index to evaluate the sensitivity and specificity of the individual and combined tests for the diagnosis of CHD. The value of the individual and combined tests for the diagnosis of CHD was analyzed. The results showed that the AUC of the combined test of HCY and Cys C was higher than that of each individual test, and its optimal cut-off value was 0.371, and the sensitivity and specificity for the diagnosis of CHD were 45.7% and 78.5%, respectively (Table 9, Figure 1).

## 4. Discussion

The umbilical cord is the only channel through which any material can be transmit ted between the mother and the fetus. Therefore, the umbilical artery blood contains a large amount of fetal–placental circulation information, and the UA S/D, PI, and RI reflect the placental resistance and the fetal intrauterine condition. In the first trimester of pregnancy, the resistance of umbilical artery blood flow is high. With the progression of pregnancy, to ensure the blood supply of normal fetal development, the placenta gradually matures, the villus increases and thickens, the resistance of the placental blood vessels decreases, and the blood flow increases. If the placenta function is poor, the placental vascular spasm, infarction, edema, and other conditions make the lumen narrow, which results in increased fetal–placental circulatory resistance and reduced umbilical artery blood flow. This affects the development and growth of the fetus [10]. Previous studies [11,12] have reported that in cases of isolated major fetal heart defects, maternal serum placental growth factor (PIGF) decreases at 11–13 weeks of gestation, which indicates that placental angiogenesis was impaired in the first trimester of pregnancy. Moreover, the overexpression of vascular endothelial growth factor A, soluble FMS-like tyrosine kinase-1 (sFlt-1), and soluble endocrine in fetuses with congenital heart disease were observed in their heart tissue and cord blood. Maternal blood PIGF levels were decreased, and sFlt-1 levels were increased at 18–37 weeks of gestation. This indicated that placental angiogenesis is affected when the fetus has congenital heart disease [10]. A study [13] reported that increased UA PI is associated with CHD in the fetus. Another study [14] reported that regardless of the type of congenital heart disease, the UA PI increased as the pregnancy progressed, which suggested that the degree of placental damage increased with the progression of pregnancy. We found that UA S/D, PI, and RI of the fetuses with CHD were higher than those of normal fetuses, and UA S/D, PI, and RI were particularly increased in the fetuses with complex CHD. UA hemodynamic changes are a good indicator of changes in placental functions [15], and the increase in UA Doppler indices in the second trimester in the CHD group in this study indicated possible placental function impairment in the second trimester of pregnancy, reflecting that CHD occurrence is associated with placental hypofunction, followed by placental ischemia and hypoxia and fetal ischemia and hypoxia, ultimately leading to adverse pregnancy outcomes. Based on previously reported studies, echocardiography is recommended in fetuses with increased UA Doppler indices in the second trimester to detect fetal heart defects as soon as possible and improve the prenatal diagnosis rate of fetal CHD. In case fetal echocardiography indicates a fetus with CHD, ultrasound should be performed regularly to measure the blood flow index of the UA to assess the growth and development of the fetus, monitor the intrauterine safety of the fetus, terminate the pregnancy on time, and ensure that newborns with CHD receive timely treatment.

This study also showed that the HCY of the simple CHD group was higher than that of the control group, and the difference was statistically significant (*p* < 0.05). The HCY level of the complex CHD group was significantly higher than that of the control group (*p* < 0.001). Previous studies [4,16,17] have shown that hyperhomocysteinemia in pregnant women may be associated with CHD occurrence in their offspring, and the present study also confirmed that high maternal HCY levels are associated with fetal cardiac malformation. HCY can pass through the placental barrier and exert cytotoxic effects, inducing excessive apoptosis in early embryonic cells, impairing placental functions, and causing embryonic malformations [18]. The abnormal differentiation of neural crest cells at high HCY concentrations may lead to neural tube defects and abnormal cardiac development [19]. If the maternal plasma HCY level can be determined in the first trimester of pregnancy, hyperhomocysteinemia can be detected as early as possible, and early screening for fetal congenital heart disease in pregnant women with hyperhomocysteinemia can be performed. Such women can be supplemented with an appropriate diet and nutrition.

Cys C is a non-glycosyl alkaline protein belonging to cysteine protease inhibitors. Previous studies showed [20,21] that Cys C can be reabsorbed and completely degraded at the proximal tubule after glomerular filtration; hence, Cys C is less affected by factors such as age, sex, weight, diet, lipid metabolism, and inflammation. Cys C levels may be associated with cardiovascular disease. Cys C impairs the cardiovascular system by affecting smooth muscle cell functions, coagulation, lipid peroxidation, and endothelial cell functions [22]. We found that Cys C levels of the subjects who were pregnant with fetuses with CHD were significantly higher than those of the normal control group (*p* < 0.05). Infants and children with CHD have increased Cys C levels, and Cys C can be used as a biomarker to predict postoperative complications of acute kidney injury after undergoing cardiac surgery [23,24]. The relationship between maternal plasma Cys C and fetal CHD has not been reported. To the best of our knowledge, we are the first to report that plasma Cys C levels of women pregnant with fetuses with CHD were higher than those of women pregnant with normal fetuses. Future studies are necessary for establishing a correlation between newborn plasma Cys C, placental Cys C, and maternal plasma Cys C levels in CHD for better understanding.

A strong positive correlation exists between Cys C levels and HCY. Cys C is a strong independent predictor of long-term all-cause death and major adverse cardiac events in elderly patients with acute myocardial infarction. The combined detection of Cys C and HCY further improves the predictive value [25]. Cys C can inhibit HCY decomposition, increase its concentration in blood, and interact with factors including plasma HCY and histones, thereby increasing the risk of hypertension and gestational diabetes in pregnant women and further increasing the risk of premature birth, miscarriage, and fetal intrauterine growth restriction [26]. The accumulated HCY in the body passes through the placental barrier; if the endothelial function of the blood vessels in the placenta is impaired, the vascular resistance in the placenta increases, which damages placental functions, affecting the blood flow motility of the UA and eventually increasing UA blood flow resistance. This study showed that increased UA Doppler indices and maternal plasma HCY and Cys C levels affected the fetuses suffering from CHD. A positive correlation was observed between maternal plasma HCY and Cys C levels and UA Doppler indices. From the clinical perspective, fetal echocardiography is a routine part of prenatal screening and is only added if a possible abnormality in heart development is detected during fetal systemic ultrasound or if there are high-risk factors for CHD, so some congenital heart diseases are not detected prenatally and are detected after birth due to presence of cardiac symptoms. The early detection of high HCY and Cys C levels in the first trimester of pregnancy or the high UA Doppler indices in the second trimester of pregnancy will help assist fetal echocardiography in fetal CHD diagnosis. This will improve the detection rate of fetal CHD, thereby increasing the rate of neonatal treatment and reducing neonatal mortality.

A limitation of our study was that not all heart malformations can be diagnosed by ultrasound in the fetus, which might lead to a possible statistical bias in our results. Fetal cardiac structure screening mainly relies on prenatal echocardiography. For those simple CHDs, such as isolated ventricular septal defect, the diagnostic accuracy of fetal echocardiography is not very high. This is mainly because some defects would close spontaneously during pregnancy or after birth. Echocardiography is not routinely performed on newborns after birth, but only newborns diagnosed with CHD in the fetal period require an echocardiogram after birth and a clinical examination of each newborn by an experienced pediatrician, including auscultation of heart murmurs and oxygen saturation of the upper and lower extremities. Another limitation of the present study was that the blood testing and UA Doppler were not performed in the same time, which could also cause some biases in the results. In the subsequent study design, we would consider performing the blood sampling and ultrasound in the same gestational age.

## 5. Conclusions

To summarize, UA S/D, PI, and RI values of the fetuses with CHD, especially complex CHD, were significantly higher than those of normal fetuses. The maternal plasma HCY and Cys C levels of women pregnant with fetuses with CHD increased significantly. Maternal plasma HCY, Cys C, and UA Doppler indices were positively correlated, which were increased in fetal CHD. The detection of increased HCY and Cys C in the first trimester of pregnancy suggests an increased risk of fetal CHD, and fetal echocardiography is required in the second trimester, indicating the screening value of increased HCY and Cys C levels in the first trimester of pregnancy. In this study, we only analyzed the relationship between maternal plasma HCY and Cys C levels in the first trimester of pregnancy, UA Doppler indices, and fetal CHD. Prospective application of these results in a validation study is still required to evaluate the clinical utility of these markers or a combination of these markers. In future large-sized studies, researchers need to investigate maternal blood indicators in the first, second, and third trimesters of pregnancy and make longitudinal comparisons to explore the correlation between various indicators and fetal CHD. A few studies on the correlation between maternal plasma Cys C and fetal CHD are available. In the future, the effect of plasma Cys C on fetal CHD can be analyzed in depth by conducting basic trials combined with prospective clinical studies, which can help to detect congenital disabilities as early as possible and reduce the incidence of adverse outcomes.

## Figures and Tables

**Table 1:** Antenatal cardiac finding in all cases of CHD.

Groups	Type of CHD	*n*
Simple CHD group	VSD	74
	PS	3
Complex CHD group	TOF	29
	TGA	16
	COA	14
	HLHS	10
	DORV	9
	HRHS	6
	TVD	6
	AVSD	5
	Vascular ring	4
	SV	6
	IAA	3
	PS + VSD	3
	PA	2
	TA	2
	PTA	2
	HCM	2
	Ebstein’s anomaly	1
Total		197

VSD, ventricular septal defect; COA, coarctation of the aorta; PS, pulmonary stenosis, TOF, tetralogy of Fallot; TGA, transposition of great arteries; HLHS, hypoplastic left heart syndrome; HRHS, hypoplastic right heart syndrome; DORV, double outlet of right ventricle; TVD, tricuspid valve dysplasia; AVSD, atrioventricular septal defect; SV, single ventricle; IAA, interruption of aortic arch; PA, pulmonary atresia; TA, tricuspid atresia; PTA, persistent truncus arteriosus; HCM, hypertrophic cardiomyopathy.

**Table 2:** Maternal characteristics of the case and control group.

Characteristics	Simple CHD Group Mean ± SD	Complex CHD Group Mean ± SD	Control Group Mean ± SD	*p*
Maternal age (y)	31.96 ± 5.41	31.29 ± 4.54	31.07 ± 3.70	>0.05
Pregestational BMI	21.84 ± 3.38	21.28 ± 3.01	21.31 ± 2.92	>0.05

**Table 3:** Comparison of the UA Doppler indices at 22–24 weeks of pregnancy.

Groups	*n*	UA S/D Mean ± SD	UA PI Mean ± SD	UA RI Mean ± SD
CHD group	197	3.40 ± 0.79	1.17 ± 0.21	0.69 ± 0.06
Simple CHD group	77	3.35 ± 0.70	1.16 ± 0.24	0.69 ± 0.06
Complex CHD group	120	3.43 ± 0.84	1.18 ± 0.19	0.70 ± 0.06
Control group	400	3.21 ± 0.57	1.12 ± 0.16	0.68 ± 0.05
*p*	Simple CHD vs. Complex CHD	0.826	0.517	0.262
	Simple CHD vs. Control group	0.288	0.055	0.254
	Complex CHD vs. Control group	0.022	0.001	0.003

**Table 4:** Comparison of HCY and Cys C levels at 12–14 weeks of pregnancy.

Groups	*n*	HCY (μmol/L) Mean ± SD	Cys C (mg/L) Mean ± SD
CHD group	197	4.56 ± 2.04	0.55 ± 0.11
Simple CHD group	77	4.35 ± 1.66	0.56 ± 0.12
Complex CHD group	120	4.69 ± 2.24	0.54 ± 0.11
Control group	400	3.78 ± 1.44	0.51 ± 0.10
*p*	Simple CHD vs. Complex CHD	0.162	0.695
	Simple CHD vs. Control group	0.005	0.007
	Complex CHD vs. Control group	<0.001	0.032

**Table 5:** Pearson correlation of UA S/D with HCY and Cys C.

Variable	r	*p*
HCY	0.157	<0.001
Cys C	0.131	0.001

**Table 6:** Pearson correlation of UA PI and HCY with Cys C.

Variable	r	*p*
HCY	0.088	0.031
Cys C	0.118	0.004

**Table 7:** Pearson correlation of UA RI and HCY with Cys C.

Variable	r	*p*
HCY	0.066	0.108
Cys C	0.118	0.004

**Table 8:** Logistic regression analysis.

Variable	B	S.E.	Wald	*p*	OR	95%CI
Maternal age	0.03	0.02	1.80	0.179	1.03	0.99–1.07
Pregestational BMI	0.02	0.03	0.53	0.469	1.02	0.97–1.08
UA S/D	0.43	0.13	10.44	0.001	1.54	1.19–2.00
UA PI	1.61	0.49	10.97	0.001	5.01	1.93–12.98
UA RI	4.24	1.54	7.55	0.006	69.55	3.38–1433.25
HCY	0.31	0.06	26.40	<0.001	1.37	1.21–1.54
Cys C	3.21	0.46	27.42	<0.001	24.75	4.75–129.10

**Table 9:** The area under the ROC.

Variable	Threshold	Sensitivity (%)	Specificity (%)	Area	S.E.	*p*	95%CI
UA S/D	3.265	51.8	59.2	0.565	0.03	0.010	0.52–0.61
UA PI	1.065	75.1	39.5	0.567	0.03	0.008	0.52–0.62
UA RI	0.685	57.9	52.2	0.564	0.03	0.011	0.52–0.61
HCY	3.850	63.5	56.0	0.626	0.02	<0.001	0.58–0.67
Cys C	0.515	60.4	54.5	0.589	0.03	<0.001	0.54–0.64
S/D + PI + RI	0.307	64.5	52.5	0.574	0.03	0.003	0.53–0.62
HCY + Cys C	0.371	45.7	78.5	0.651	0.02	<0.001	0.60–0.70

## Data Availability

The data presented in this study are openly available in FigShare at 10.6084/m9.figshare.21300045.
